# Sugar-sweetened beverage purchases in urban Peru before the implementation of taxation and warning label policies: a baseline study

**DOI:** 10.1186/s12889-022-14762-w

**Published:** 2022-12-20

**Authors:** Caitlin M. Lowery, Lorena Saavedra-Garcia, Francisco Diez-Canseco, María Kathia Cárdenas, J. Jaime Miranda, Lindsey Smith Taillie

**Affiliations:** 1 Department of Nutrition, University of North Carolina at Chapel Hill, Chapel Hill, NC USA; 2 CRONICAS Center of Excellence in Chronic Diseases, Universidad Peruana Cayetano Heredia, Lima, Peru; 3 Carolina Population Center, University of North Carolina at Chapel Hill, Chapel Hill, NC USA; 4 School of Medicine, Universidad Peruana Cayetano Heredia, Lima, Peru; 5 The George Institute for Global Health, UNSW, Sydney, NSW Australia; 6 Gillings School of Global Public Health, University of North Carolina at Chapel Hill, 123 W Franklin St, Ste 2107, Chapel Hill, NC 27516 USA

**Keywords:** Nutrition Policy, Socioeconomic Factors, Sugar-sweetened Beverages, Taxation, Obesity, Peru

## Abstract

**Background:**

Sugar-sweetened beverage consumption is associated with obesity and chronic disease. In 2018, Peru increased the tax on high-sugar beverages (≥6 g of sugar per 100 mL) from 17 to 25%, yet little is known about pre-existing beverage trends or demographic characteristics associated with purchases in the country. The aim of this study was to explore beverage purchasing trends from 2016 to 2017 and examine variation in purchase volume by sociodemographic characteristics among urban households in Peru.

**Methods:**

This study used monthly household purchase data from a panel of 5145 households from January 2016–December 2017 from Kantar WorldPanel Peru. Beverage purchases were categorized by type and tax status under the 2018 regulation (untaxed, lower-sugar taxed, high-sugar taxed). To assess beverage purchasing trends, per-capita volume purchases were regressed on a linear time trend, with month dummies for seasonality and clustered standard errors. Mean volume purchases by beverage tax status (total liters purchased per month), overall and by key demographic characteristics (education, socioeconomic status, and geographic region), were calculated. Mean volume by beverage type was assessed to identify the largest contributors to total beverage volume.

**Results:**

The trends analysis showed a decline in total beverage volume of − 52 mL/capita/month (95% CI: − 72, − 32) during the 24-month study period. Over 99% of households purchased untaxed beverages in a month, while > 92% purchased high-sugar taxed beverages. Less than half of all households purchased low-sugar taxed beverages in a month and purchase volume was low (0.3 L/capita/month). Untaxed beverage purchases averaged 9.4 L/capita/month, while households purchased 2.8 L/capita/month of high-sugar taxed beverages in 2017. Across tax categories, volume purchases were largest in the high education and high socioeconomic (SES) groups, with substantial variation by geographic region. The highest volume taxed beverage was soda (2.3 L/capita/month), while the highest volume untaxed beverages were milk and bottled water (1.9 and 1.7 L/capita/month, respectively).

**Conclusions:**

Nearly all households purchased high-sugar taxed beverages, although volume purchases of taxed and untaxed beverages declined slightly from 2016 to 2017. Households with high SES and high education purchased the highest volume of taxed beverages, highlighting the need to consider possible differential impacts of the tax policy change by sub-population groups.

**Supplementary Information:**

The online version contains supplementary material available at 10.1186/s12889-022-14762-w.

## Background

Sugar-sweetened beverage (SSB) consumption has been implicated in the obesity epidemic and growing burden of chronic disease worldwide [1–3]. In the past several decades, Latin America has experienced a nutrition transition [4], shifting away from traditional dietary patterns and toward purchases of ultra-processed foods [5, 6], and is now among the highest SSB-consuming regions in the world [7]. In Peru, as in other Latin American countries, undernutrition persists, particularly in rural and low-income communities, although the prevalence has declined, while obesity and overweight have increased [8], a phenomenon known as the double burden of malnutrition [9].

Estimates of SSB consumption in Peru vary by age, gender and beverage type, ranging from 0.42 and 0.39 servings [one serving is 8 oz or 237 mL] per day among men and women aged ≥80 years, respectively, to 1.52 and 1.39 servings per day among men and women aged 20–29 years in a study of global SSB consumption [7], to 1.3 servings/day of homemade and 0.7 servings/day of ready-to-drink (RTD) SSBs for men and 1.1 servings/day of homemade SSBs and 0.3 servings/day of RTD SSBs for women based on data from the 2017–2018 Peruvian National Health Survey [10]. Consumption of SSBs has been linked to overweight and obesity, type II diabetes, and cardiovascular disease [11–13]. Given these health risks, it is essential to understand SSB purchasing patterns in the Peruvian context, including what sociodemographic characteristics are associated with SSB purchases and what types of SSBs households are purchasing.

Recent policy developments underscore the need for a more complete awareness of SSB purchasing habits in Peru. In the last decade, policies to curb consumption of SSBs, such as taxation and front-of-package warning label requirements, have become increasingly popular across Latin America, including in Peru. A meta-analysis of SSB taxes around the globe found that a 10% tax on SSBs was associated with a 10% decrease in purchasing or consumption [14]. Peru implemented an increase to a pre-existing tax on beverages with added sugar in May 2018, raising the tax on high-sugar beverages (≥6 g of sugar per 100 mL) from 17 to 25% [15]. The initial tax on beverages among other goods (*Impuesto Selectivo al Consumo*) was passed in 1999 as a revenue-generating measure, rather than a public health initiative, and until 2006, included bottled water as well as carbonated beverages [16]. The cut-off for the higher tax tier was aligned with the “high sugar” threshold for the mandatory front-of-package label (FOPL) policy implemented in 2019, which required black octagons on products containing ≥6 g of sugar per 100 mL [17]. In order to evaluate the effectiveness of the tax increase and FOPL policy, it is necessary to first understand SSB purchasing trends and in particular, which groups purchase high amounts of SSBs, in order to identify which groups are most likely to respond to these policies.

The primary objective of this study was to assess trends in beverages purchasing among urban households in Peru from 2016 to 2017, prior to the tax increase on high-sugar beverages. This study also aimed to estimate the percentage of households purchasing high-sugar taxed (≥6 g of sugar/100 mL), lower-sugar taxed (< 6 g of sugar/100 mL) and tax-exempt beverages in a month, and the monthly volume of household beverage purchases, overall and by key sociodemographic characteristics. Finally, the study sought to identify the beverage types contributing the largest volume to taxed and untaxed beverage purchasers. This analysis will provide important context for future evaluations of the impact of two recent policy changes (i.e., the beverage tax increase and FOPL requirement), which may have differential effects based on education, income, and geographic region, and will offer insight into household beverage purchasing behaviors in Peru.

## Methods

This study used anonymized monthly household purchase data from January 2016 through December 2017 from Kantar WorldPanel Peru. A panel of 3800 households was recruited through stratified random sampling. Households that left the panel were replaced by randomly selected households with similar demographic characteristics, after completing a three-month run-in period for quality assurance. With replacement, the analytic sample comprised 5145 unique households, with an average of 18 months of follow-up (median: 23), providing 90,654 household-month observations. Households were recruited from 14 major cities in six geographic regions of the country (Lima, central coast, northern coast, southern coast, the Andean highlands, and the Amazon). Kantar WorldPanel Peru provides monthly household sampling weights to ensure the panel’s representativeness of 67% of the urban population of Peru. An area is considered urban if it has at least 100 dwellings in a contiguous group or is a district capital, and it has at least 2000 inhabitants [18]. Kantar WorldPanel Peru excludes households with a potential conflict of interest related to products studied and households who do not meet minimum purchasing standards (e.g., not purchasing any items from a 15-category “basket” of basic goods in a month).

Trained data collectors visited participating households weekly to scan barcodes of all packaged food and beverage items purchased for at-home consumption, using standardized codebooks for bulk products and items without barcodes. Panelists were instructed to save all receipts and empty containers to be scanned. The dataset contains item-level information including barcode, product name, brand, description, volume, price per unit, and date of purchase. Product-level data were used to link beverage purchases to nutrition facts panel (NFP) data based on barcode, brand, and product description, as in previous studies [19].

﻿NFP data were obtained from product photographs collected in grocery stores by a team of Peruvian research assistants in 2018, which has been described elsewhere [20] and managed using REDCap electronic data capture tools hosted at UNC [21]. If no collected NFP data were available for a purchased product, it was linked to nutrition facts panel data from Mintel Latin America. After the linkage, all beverages in the dataset were categorized by trained nutritionists according to beverage type and tax status under the 2018 regulation [15] (untaxed, lower-sugar taxed, high-sugar taxed) (Supplemental Table 1). High-sugar taxed beverages, such as soda, were defined as drinks containing ≥6 g of sugar per 100 mL, while lower-sugar taxed beverages like diet soda were defined as drinks containing < 6 g of sugar per 100 mL. Beverages containing no added sweeteners, such as bottled water, 100% fruit juice, plain milk, as well as drinkable yogurt, infant formula, and powdered fruit-flavored drink mixes, were exempt from the regulation.

Beverage types included water, milk, regular soda, diet soda, fruit juice drinks (juices or nectars containing < 100% fruit juice), *refrescos* (fruit-flavored drinks), dairy drinks (flavored or sweetened evaporated or condensed milk drinks), coffee, tea, 100% fruit juice, and sports and energy drinks. Beverage purchases were reported per month by beverage type. Volume (mL) purchases for each beverage type were divided by household size to calculate per-capita volume purchases. Each observation therefore represents one household’s per-capita beverage purchases in a month.

Demographic data was collected upon enrollment in the study and updated annually. Key demographic characteristics reported included socioeconomic status (SES), head of household educational attainment, region, number of children under 13 years of age and household size. Head of household was defined the person who lives in the home and generates the most income for the household and/or makes the financial choices of the family. Head of household education was categorized into three bins for analysis (less than high school, completed high school, and more than high school) from ten original categories.

Household SES was calculated from an assets index and key sociodemographic characteristics. This measure was developed by the Peruvian Association of Market Research Firms (APEIM) and has been applied to data from the Peruvian National Household Survey (ENAHO) to provide population-level SES estimates [22] and has been used in prior studies [23, 24]. Specifically, household SES was categorized from A (high) to E (low), based on ownership of items such as washing machines and cars, as well as living conditions like floor material and bathroom type (indoor/outdoor), and sociodemographic characteristics like education and insurance status. Because the proportion of households in category A was small (< 5% of households), these households were combined with category B for analysis.

### Statistical analysis

To assess trends in beverage volume purchases, separate weighted OLS regressions of total volume, untaxed volume, lower-sugar taxed volume, and high-sugar taxed volume purchases per capita per month on a linear time trend (i.e., treating month as a continuous variable with range 1–24) were run. Month dummies [1–12] were included to account for seasonality and standard errors were clustered at the household-level. Plots generated from weighted OLS regressions of total volume purchases and volume purchases by tax status in L per capita per month treating month as a factor variable were visually inspected to understand seasonal variation.

The percentage of households purchasing each beverage type in a month and the mean unadjusted volume of purchases by beverage tax status, overall and by key demographic characteristics (region, education, and SES) were calculated, using survey weights. Weighted, mean unadjusted volume purchases by beverage type for high-sugar taxed, lower-sugar taxed and untaxed beverages, overall and by key sociodemographic characteristics, were estimated to explore the beverage types contributing the largest volume to beverage purchases by taxation status. Volume purchases among households that purchased any beverages with a particular tax status (i.e., excluding those who made no purchases in a tax category in a month) were also assessed. Volume purchases per capita of each beverage type, independent of tax status (i.e., combining both taxed and untaxed *refrescos* into a single category), were also assessed. Because the pattern of results was similar across years, we report results from 2017 only (2016 results are available in Supplemental Tables 4, 5, and 6). As a sensitivity analysis, we also conducted the analyses without survey weights (available in Supplemental Table 3). Furthermore, because young children may consume a lower volume than older children or adults, we performed two sensitivity analyses excluding a) children under 2 and b) children under 5 from the total number of household members when calculating the volume per capita (results available in Supplemental Tables 7 and 8).

All analyses used cluster-robust standard errors at the household level to account for repeated measures. Analyses were conducted using Stata 16 (College Station, TX, USA).

## Results

Sample characteristics were comparable in 2016 and 2017. Nearly one-third of households were from the Lima metropolitan area and 27% were from the northern coast, with smaller numbers of participating households from the southern coastal, Andean highlands, central coastal and Amazon regions (Table 1). About one-fifth of the household heads had less than a high school education in 2016 and 2017. The majority of households (~ 69%) were in the middle (C) or lower-middle (D) SES groups. About 18% were in the highest SES categories (A/B) and 13–14% were in the lowest category (E). Mean household size was 4.4 individuals (SD: 1.8) in 2016 and 4.1 individuals (SD: 1.7) in 2017, with an average of 1.3 children aged ≤12 years (SD: 1.1) in 2016 and 1.2 children (SD: 1.1) in 2017.

**Table 1:** Demographic characteristics of the sample

	2016 (***N*** = 4367)	2017 (***N*** = 4488)
Demographics	N (%)	N (%)
**Region**		
Lima	1299 (29.7)	1287 (28.7)
Central Coast	430 (9.8)	467 (10.4)
Northern Coast	1174 (26.9)	1220 (27.2)
Southern Coast	562 (12.9)	577 (12.9)
Amazon	346 (7.9)	364 (8.1)
Highlands	556 (12.7)	573 (12.8)
**Education**^**a, b**^		
Less than HS	888 (20.3)	887 (19.8)
HS Graduate	1851 (42.4)	1950 (43.5)
More than HS	1628 (37.3)	1648 (36.7)
**SES**^**c**^		
A/B (High)	788 (18.0)	803 (17.9)
C	1461 (33.5)	1475 (32.9)
D	1541 (35.3)	1598 (35.6)
E (Low)	577 (13.2)	612 (13.6)
	**Mean (SD)**	**Mean (SD)**
Household Size	4.4 (1.8)	4.1 (1.7)
Children ≤12	1.3 (1.1)	1.2 (1.1)

^a^ Education was grouped into three categories: did not complete high school (secondary school), graduated high school, and completed technical school, university or graduate school

^b^ Three households were missing education in 2017

^c^ SES was determined based on an assets index developed by the Peruvian Association of Market Research Firms (APEIM). Categories A and B were combined because few households were in the highest SES (A) category (2016: 4.5%; 2017: 4.4%)

### Beverage purchasing trends

**Fig. 1:**
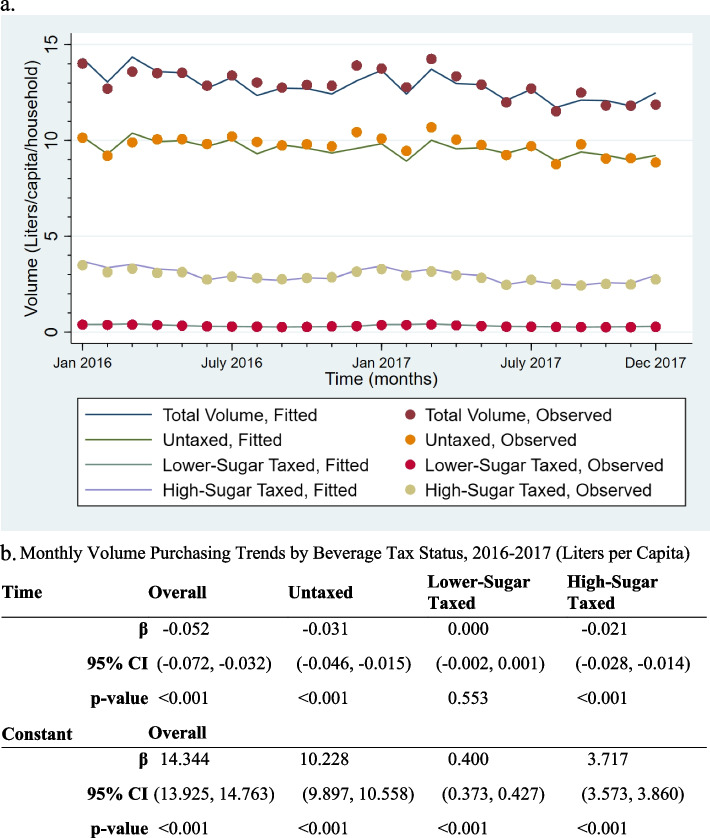
Monthly Volume Purchasing Trends by Beverage Tax Status, 2016–2017 (Liters per Capita). Results are from separate weighted OLS regressions of total volume purchases, untaxed beverage volume purchases, lower-sugar taxed volume purchases, and high-sugar taxed volume purchases (L) per capita per month on a linear time trend with cluster-robust standard errors at the household level. The model also included month dummies (1-12) to account for seasonality (omitted from output). Time is a continuous variable (1-24). β is the beta coefficient, 95% CI is the 95% confidence interval, and p is the *p*-value for the statistical significance of the beta coefficient

The trends analysis revealed a decline in purchasing across all beverage types, with an overall decline in total beverage volume of − 52 mL (95% CI: − 71.7, − 32.4), or 1.8 oz., per capita per month across the time period **(**Fig. 1). Untaxed beverage purchase volume decreased by − 31 mL (95% CI: − 46.3, − 15.3) per capita per month, while high-sugar taxed beverage volume purchases declined by − 21 mL (95% CI: − 27.5, − 14.1) per capita per month. Lower-sugar taxed beverage purchases had no statistically significant change over time (− 0.4 mL [95% CI: − 1.7, 0.9]).

**Fig. 2:**
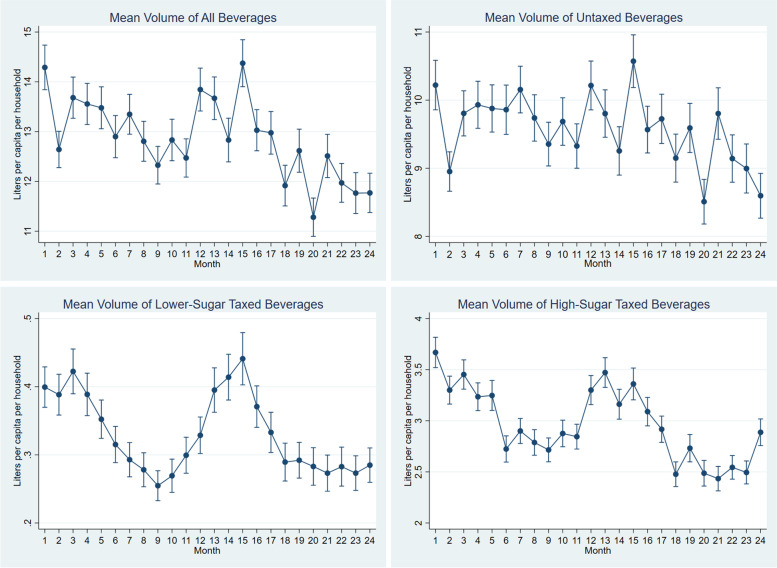
Weighted Mean Monthly Volume Purchases, Overall and by Taxation Status Please note that the different scales of the graphs, which vary by beverage type, in order to show the seasonal variability of purchasing trends

Beverage purchases followed seasonal trends (Fig. 2). Volume purchases were highest in December through March, which corresponds to the warmer summer months. The trend was less pronounced for untaxed beverages than for high-sugar and lower-sugar taxed beverages.

### Percentage of household purchasers and mean beverage volume by future tax status

Purchasing patterns were similar across sociodemographic characteristics in 2016 and 2017, although volume purchases decreased in 2017, in line with the results of the trends analysis. For simplicity, Tables 2, 3 and 4 include results for 2017 only (2016 results are available in the Supplementary Material). Nearly all households in the weighted sample (99.8%) purchased untaxed beverages in a month in 2017 (Supplemental Table 2). High-sugar taxed beverages were purchased by 92% of households, while lower-sugar taxed beverages were less popular, purchased by 44.4% of households in a given month. Untaxed beverages made up the majority of all beverage purchases; mean per capita purchases of untaxed beverages were 9.4 (95% CI: 9.1, 9.7) L/month (Table 2). Per capita purchases of high-sugar taxed beverages averaged 2.8 (2.7, 2.9) L per month. The volume of lower-sugar taxed beverages was low (0.3 L/capita/month). After excluding children under 2 and children under 5 from the per capita calculation in sensitivity analyses (see Supplemental Tables 7 and 8), results followed the same patterns, with slightly higher volume per capita purchases. Unweighted models also produced similar results (Supplemental Table 3), as mean per capita purchases of untaxed beverages averaged 9.5 (9.3, 9.8) L/month, while mean high-sugar taxed beverage volume was 2.8 (2.7, 2.8) L/month.

**Table 2:** Weighted mean monthly purchase volume by beverage tax status (Liters per Capita) in 2017

Beverage Type	Untaxed	Tax Tier 1 (Lower-Sugar)	Tax Tier 2 (High-Sugar)
	Mean (95% CI)	Mean (95% CI)	Mean (95% CI)
**Overall**	9.4 (9.1, 9.7)	0.3 (0.3, 0.3)	2.8 (2.7, 2.9)
**Region**			
Lima	9.8 (9.4, 10.2)	0.3 (0.3, 0.4)	3.0 (2.8, 3.1)
Central Coast	8.3 (7.8, 8.7)	0.4 (0.3, 0.4)	3.0 (2.7, 3.3)
Northern Coast	7.9 (7.4, 8.3)	0.3 (0.3, 0.4)	1.7 (1.5, 1.8)
Southern Coast	8.1 (7.6, 8.6)	0.1 (0.1, 0.2)	3.4 (3.2, 3.7)
Amazon	18.6 (16.2, 21.0)	0.3 (0.3, 0.4)	2.9 (2.6, 3.1)
Highlands	8.3 (7.7, 9.0)	0.3 (0.3, 0.4)	3.8 (3.4, 4.1)
**Education**^**a,b**^			
Less than HS	8.2 (7.6, 8.8)	0.3 (0.3, 0.4)	2.7 (2.5, 2.9)
HS Graduate	8.4 (8.1, 8.8)	0.3 (0.3, 0.3)	2.6 (2.5, 2.8)
More than HS	11.1 (10.6, 11.7)	0.4 (0.3, 0.4)	3.2 (3.0, 3.4)
**SES**^**c**^			
A/B (High)	13.1 (12.2, 13.9)	0.4 (0.4, 0.5)	3.6 (3.3, 4.0)
C	9.6 (9.1, 10.0)	0.3 (0.3, 0.3)	2.9 (2.7, 3.0)
D	8.1 (7.7, 8.5)	0.3 (0.3, 0.3)	2.5 (2.3, 2.6)
E (Low)	6.9 (6.3, 7.5)	0.3 (0.3, 0.3)	2.5 (2.3, 2.8)

Mean volume by tax status, overall and by key demographic characteristics, was calculated using sample weights and standard errors clustered at the household-level

^a^ Education was grouped into three categories: did not complete high school (secondary school), graduated high school, and completed technical school, university or graduate school

^b^ Three households were missing education in 2017

^c^ SES was determined based on an assets index developed by the Peruvian Association of Market Research Firms (APEIM). Categories A and B were combined because few households were in the highest SES (A) category (2017: 4.4%)

**Table 3:** Weighted mean monthly purchase volume of the top three beverage types by taxation status (Liters per Capita per Household) in 2017

	Mean (95% CI) L/capita/ Household	Percent Purchasers (%) (95% CI)	Mean (95% CI) L/capita/ household (Among Purchasers)
Untaxed			
*Water, plain*	1.7 (1.6, 1.8)	59.8 (58.6, 61.0)	2.9 (2.7, 3.1)
*Milk, plain*	1.9 (1.8, 1.9)	85.5 (84.8, 86.3)	2.2 (2.1, 2.3)
*Dairy drinks*^*a*^	1.1 (1.0, 1.1)	59.6 (58.3, 60.8)	1.8 (1.7, 1.9)
Lower-sugar Taxed			
*Regular soda*	0.1 (0.1, 0.2)	21.0 (20.0, 21.9)	0.7 (0.7, 0.7)
*Diet soda*	0.1 (0.1, 0.1)	11.6 (10.9, 12.3)	0.6 (0.5, 0.7)
*Sports drinks*	0.1 (0.1, 0.1)	22.0 (21.1, 23.0)	0.4 (0.4, 0.4)
High-sugar Taxed			
*Regular soda*	2.1 (2.1, 2.2)	85.4 (84.6, 86.2)	2.5 (2.4, 2.6)
*Fruit juice drinks*^*b*^	0.2 (0.2, 0.3)	44.5 (43.3, 45.7)	0.6 (0.5, 0.6)
*Refrescos*^*c*^	0.2 (0.2, 0.2)	29.7 (28.6, 30.7)	0.7 (0.7, 0.8)

Mean volume for the three highest volume beverage types in each tax status category in 2017 was calculated using sample weights and standard errors clustered at the household-level. Percent purchasers reflects the mean percentage of the sample purchasing each beverage type in a month

^a^ Dairy drinks are milk-based drinks that contain other ingredients such as oil (vegetable, palm), sugar, honey, cereal, or flour

^b^ Fruit juice drinks include nectars and juices containing < 100% fruit juice

^c^ *Refrescos* are fruit-flavored drinks, containing water, sugar, and flavoring

**Table 4:** The three highest volume beverage types by Region, SES, and Education in 2017

	Beverage #1		Beverage #2		Beverage #3	
	Bev type	Per capita mean (95% CI)	Bev type	Per capita mean (95% CI)	Bev type	Per capita mean (95% CI)
**Total**	Soda (reg)	2.3 (2.2, 2.4)	Milk (plain)	1.9 (1.8, 1.9)	Water	1.7 (1.6, 1.8)
**Region**						
Lima	Soda (reg)	2.4 (2.2, 2.5)	Milk (plain)	1.9 (1.8, 2.0)	Dairy drinks^d^	1.4 (1.3, 1.5)
Central Coast	Soda (reg)	2.5 (2.2, 2.7)	Milk (plain)	1.9 (1.8, 2.1)	Water	1.5 (1.3, 1.8)
Northern Coast	Milk (plain)	1.7 (1.6, 1.8)	Water	1.7 (1.4, 1.9)	Soda (reg)	1.5 (1.4, 1.6)
Southern Coast	Soda (reg)	2.7 (2.5, 3.0)	Milk (plain)	1.9 (1.8, 2.0)	Dairy drinks^d^	0.8 (0.7, 0.9)
Amazon	Water	12.2 (10.2, 14.2)	Soda (reg)	2.5 (2.3, 2.7)	Milk (plain)	1.7 (1.4, 1.9)
Highlands	Soda (reg)	2.6 (2.4, 2.9)	Milk (plain)	2.2 (2.0, 2.4)	Dairy drinks^d^	1.1 (0.9, 1.3)
**Education**^**a,b**^						
Less than HS	Soda (reg)	2.3 (2.1, 2.5)	Milk (plain)	1.8 (1.6, 1.9)	Water	1.4 (1.1, 1.6)
HS Graduate	Soda (reg)	2.1 (2.0, 2.2)	Milk (plain)	1.8 (1.7, 1.9)	Water	1.4 (1.3, 1.6)
More than HS	Soda (reg)	2.5 (2.3, 2.7)	Water	2.2 (2.0, 2.5)	Milk (plain)	2.1 (1.9, 2.2)
**SES**^**c**^						
AB	Soda (reg)	2.8 (2.5, 3.1)	Water	2.7 (2.3, 3.1)	Milk (plain)	2.2 (2.1, 2.4)
C	Soda (reg)	2.4 (2.2, 2.5)	Milk (plain)	1.9 (1.8, 2.0)	Water	1.5 (1.4, 1.7)
D	Soda (reg)	2.0 (1.9, 2.1)	Milk (plain)	1.8 (1.7, 1.9)	Water	1.4 (1.2, 1.6)
E	Soda (reg)	2.0 (1.8, 2.2)	Milk (plain)	1.6 (1.4, 1.7)	Water	1.4 (1.0, 1.8)

Mean volume for the highest volume beverage types (independent of tax status), overall and by key demographic characteristics, for 2017 was calculated using sample weights and standard errors clustered at the household-level

^a^ Education was grouped into three categories: did not complete high school (secondary school), graduated high school, and completed technical school, university or graduate school

^b^ Three households were missing education in 2017

^c^ SES was determined based on an assets index developed by the Peruvian Association of Market Research Firms (APEIM). Categories A and B were combined because few households were in the highest SES (A) category (4.5%)

^d^ Dairy drinks are milk-based drinks that contain other ingredients such as oil (vegetable, palm), sugar, honey, cereal, or flour

While the proportion of households purchasing each beverage type did not vary by SES or education, the high (A/B) SES category purchased the largest volume of beverages across taxation categories, although the difference was most pronounced for untaxed beverages; high SES households purchased 13.1 (95% CI: 12.2, 13.9) L/capita/month compared to low SES households’ 6.9 (95% CI: 6.3, 7.5) L/capita/month of untaxed beverages in 2017. Untaxed and high-sugar beverage purchases followed an SES gradient, which leveled off for groups D and E in the case of high-sugar beverages. Similarly, households in which the household head had more than a high school education purchased the largest volume in each beverage tax category, although few differences were observed between the middle and low education groups across tax categories.

Purchasing patterns by region were heterogenous. Per capita purchases of untaxed beverages per month in the Amazon region were nearly double those of any other region in 2017 (18.6 L [95% CI: 16.2, 21.0]), driven by purchases of water. Conversely, the largest volume of high-sugar taxed beverage purchases occurred in the Andean highlands with a mean of 3.8 L/capita/month (95% CI: 3.4, 4.1) and the southern coast with a mean of 3.4 L/capita/month (95% CI: 3.2, 3.7), although regional differences were less distinct for high-sugar taxed beverages than untaxed beverages.

### Percentage of household purchasers and mean beverage volume by beverage type

Of untaxed beverages, plain milk was the most commonly purchased, with ~ 85% of households purchasing it in a given month, and had the highest volume purchased in the full sample (Table 3). Dairy drinks and plain bottled water were also commonly purchased, with approximately 60% of households purchasing each beverage type.

The beverage type with the highest mean volume per capita per month in the full sample was high-sugar regular soda, at 2.1 L (95% CI: 2.1, 2.2) in 2017. Approximately 85% of households purchased high-sugar regular soda in a month in 2017. The volume of high-sugar regular soda purchased (among purchasers) was 2.5 L (95% CI: 2.4, 2.6) per capita per month in 2017. Fewer than half of all households purchased any lower-sugar taxed beverage in a month, of which regular soda containing < 6 g of sugar per 100 mL had the highest volume.

### Top contributors to beverage volume purchases by SES, education, and region

Table 4 reports the mean volume purchases of the top three beverage types by volume purchased, irrespective of taxation status. Regular soda was the most purchased beverage by volume across all levels of SES and education, although the volume was highest in the high SES (AB) and high education households. Additionally, households with high SES and high education purchased more water than plain milk, but this pattern was reversed for all other SES and education categories. Top contributors to beverage volume purchased varied by region. Regular soda was the top contributor for four of the six regions, followed by plain milk. However, water purchases predominated in the Amazon region and the highest volume beverage on the northern coast was plain milk.

## Discussion

This study set out to examine beverage purchases among urban households in Peru prior to the implementation of a tax increase on sugary drinks. Our results indicate that per-capita beverage purchase volume declined marginally for untaxed and high-sugar taxed beverages from 2016 to 2017. Purchase volume varied by season, with higher volume purchases from January to March, which corresponds to the Peruvian summer. Purchases of high-sugar taxed beverages averaged 2.8 L/capita/month (95% CI: 2.7, 2.9) in 2017 (92 mL/capita/day), with variation by geographic region (from 1.7 L/capita/month in the northern coast to 3.8 L/capita/month in the Andean highlands). This is comparable to a prior study’s estimates of purchases of “high in” beverages in Chile (128 mL/capita/day [SE: 1.8]) before the implementation of a front-of-package warning label law [19], although lower than estimates of pre-tax purchases of beverages taxed under Mexico’s 2014 SSB law (214 mL/capita/day [95% CI: 212.3, 215.6]) [25].

The prevalence of purchasing any high-sugar taxed beverage (92%) and any untaxed beverage (> 99%) in a month was extremely high, with limited variation across sociodemographic groups. However, estimated mean volume purchases differed by region, particularly for purchases of untaxed beverages. Specifically, in the Amazon region, purchases of water were notably higher than in any other part of the country. Limited access to safe drinking water is one possible explanation. The Amazon region has lower access to treated, piped drinking water than other regions of the country [26, 27], as well as a higher proportion of households without access to a toilet or latrine [28], which could motivate households to purchase potable bottled water. Temperature could also play a role, as the Amazon is warmer than other regions of Peru [29] and environmental temperature and water intake are linked [30], although the northern coast also experiences high temperatures but had similar mean volume purchases of water to other regions.

While mean purchases of high-sugar taxed beverages were more homogenous than purchases of untaxed beverages, the Andean highlands and the southern coast purchased the largest volume of high-sugar taxed beverages. This partially aligns with results of a prior study of SSB consumption using national data, which found that consumption of homemade SSBs was the highest in the Andean highlands, while the highest mean consumption of ready-to-drink SSBs occurred in the southern and central coasts [10]. In this study, the southern coast had the highest proportion of high SES households outside of Lima, which may have contributed to the larger volume of high-sugar beverage purchases. However, the Andean highlands had relatively few high SES households, but purchased a high volume of SSBs. Qualitative research to explore the drivers of high-sugar beverage purchases in the Andean region is needed. Nonetheless, our findings may help identify populations at high-risk of cardiometabolic disease and inform policies aimed at reducing SSB purchases.

With respect to education and SES, we found that the high education and high SES groups purchased the largest volume of both taxed and untaxed beverages. Similar to this study’s results, an analysis of trends in consumption of healthy and unhealthy foods in Peru using nationally representative data found that both households with higher SES and more than secondary education had higher energy consumption from unhealthy foods than other households, although they noted increasing trends across most levels of education and SES between 2001 and 2018 [8]. Prior research in urban populations has also shown an inverse relationship between Dietary Quality Score and SES in Peru [31]. On the other hand, a study of ready-to-drink and homemade SSB consumption found that individuals who completed secondary school (the middle education category) consumed greater quantities of both types of SSBs than those with less education, but had no statistically significant differences in consumption of either beverage type from the higher education group [10]. Taken together, this suggests that households with higher SES and education currently consume more unhealthy products, although this may be changing as Peru progresses through the obesity transition [32]. The obesity transition model, as proposed by Jaacks et al., suggests that as countries progress through the stages of the obesity transition, the burden of overweight and obesity generally shifts from higher SES to lower SES populations, as rates of overweight and obesity rise among lower SES groups and plateau among high SES groups [32], likely driven by changes in dietary patterns. Notably, while purchasing and consumption are driven by a complex set of factors including purchasing power, marketing, access, and cultural acceptability, in this study we were not able to determine which factors have largest influence based on the available data. Given the health implications of high consumption of sugary drinks, research to identify drivers of SSB purchases and interventions to reduce consumption is warranted.

The observed variability in purchases of taxed and untaxed beverages has implications for the impact of the 2018 and 2019 changes to Peru’s beverage tax policy on household beverage purchases. Since high SES households bought a large volume of high-sugar taxed beverages in this sample, and price elasticities of SSBs and soft drinks may be lower for high-income households compared to low-income households [33, 34], the policy changes may not be associated with large reductions in purchases, if the price increase due to the tax is not sufficient to reduce demand among high SES consumers. However, the variable used in this study, SES, reflects household assets and education, rather than income, and though SES is correlated with income, it may have different implications for price elasticity (i.e., the degree to which an increase in price reduces demand). Furthermore, other beverage tax evaluations have found larger changes in purchases among high SES households [35, 36]. Specifically, in Chile, decreases in purchases of high-sugar taxed beverages post-tax implementation were driven by reductions in purchases among high and middle SES households, which had higher pre-tax purchases of taxed beverages [35, 36]. In contrast, low SES households in Mexico had the largest absolute and relative reductions in purchases of taxed beverages following the Mexican SSB tax [37], but they also purchased more unhealthy beverages than high SES households before the tax’s implementation [38], which was not the case in Peru in this pre-tax study. While evaluations of beverage taxes in other Latin American countries may provide useful points of comparison, it is important to recognize the varied political, social, cultural and economic context in which each policy has been implemented, as well as differences in tax structure and implementation, which may lead to different behavioral and industrial responses [39]. Thus, it will be important to assess potential differential responses to Peru’s SSB tax policy changes by sub-population groups, to understand the implications for health equity.

A key strength of this study is the use of data collected throughout the year, which reflects households’ usual purchasing habits and allows for examination of seasonality. Data collectors visited households frequently (once per week), which may facilitate the capture of items purchased as participants do not need to retain the packaging for long periods of time. The use of barcode scanners enabled the researchers to match items purchased with specific nutrition, brand, and volume information. Linking products to nutrition facts panel data also facilitated the classification of products according to taxation status under the 2018 beverage tax increase. Another strength is the panel design of the study as the same households were followed over time. Finally, the weights provided by Kantar WorldPanel Peru allow the production of estimates representative of the urban population of Peru.

This study has some limitations. First, this dataset is based on scanned household purchases for at-home consumption. Purchases may be under-reported if households did not scan all items they bought. Furthermore, this data does not include beverages consumed outside of the home (e.g., at restaurants), which may be a significant source of sugary drink consumption. Second, because powdered mixes and concentrates were reconstituted to estimate the liquid volume of beverages purchased, water may be double counted in some cases (i.e., if bottled water was used to prepare beverages from the mixes or concentrates). Third, the analysis only includes data from 2016 and 2017. Between January and March 2017, the northern and part of the central coast of Peru experienced heavy rains and flooding associated with “El Niño”, which may have caused supply chain disruptions [40]. Such disruptions may have resulted in temporary price increases and may have affected household purchases [40], although no major changes in beverage purchase volume were observed during that period in this study. Furthermore, we do not account for other potential macroeconomic changes in the country over the 2016–2017 period that may have affected household purchasing capacity or beverage prices. Additionally, this study classifies beverages based on their taxation status under the 2018 regulation, which provides useful context for future evaluations of the policy. However, “untaxed” is not synonymous with healthy; products like drinkable yogurts or powdered drink mixes are exempt from the tax but may still contain high amounts of sugar. Finally, the dataset only includes urban households and thus, it is not possible to make inferences about rural beverage purchases. Although most Peruvians live in urban areas, from a health equity standpoint, it is crucial to understand rural purchasing patterns, as rural households have fewer economic resources [41], higher prevalence of the dual burden of malnutrition [42], and lower access to safe drinking water [27, 28].

## Conclusion

Nearly all households purchased untaxed and high-sugar taxed beverages in a month, although purchase volume varied by SES, education, and region, and declined slightly over the two-year study period. Regular soda was the highest volume beverage type, with a monthly mean of 2.3 L/capita/household. Households with high SES and high education purchased the highest volume of taxed beverages, which may have implications for the impact of the 2018 tax policy change on purchases.

## Supplementary Information

**Additional file 1: Supplemental Fig. 1.** Timeline of the Implementation of Nutrition-Related Polices and Modifications. **Supplemental Table 1.** Beverage Types by Tax Status (under the 2018 regulation). **Supplemental Table 2.** Weighted Percentage of Households Purchasing Any Beverage by Tax Status in a Month. **Supplemental Table 3.** Unweighted Mean Monthly Purchase Volume by Beverage Tax Status (Liters per Capita per Household). **Supplemental Table 4.** Weighted Mean Monthly Purchase Volume by Beverage Tax Status (Liters per Capita per Household) in 2016. **Supplemental Table 5.** Weighted Mean Monthly Purchase Volume of the Top Three Beverage Types by Taxation Status (Liters per Capita per Household) in 2016. **Supplemental Table 6.** The Three Highest Volume Beverage Types by Region, SES, and Education in 2016. **Supplemental Table 7.** Weighted Mean Monthly Purchase Volume by Beverage Tax Status in Liters per Capita^a^ per Household (Excluding Children Aged < 2 Years). **Supplemental Table 8.** Weighted Mean Monthly Purchase Volume by Beverage Tax Status in Liters per Capita^a^ per Household (Excluding Children Aged < 5 Years).

## Data Availability

The data that support the findings of this study are available from Kantar WorldPanel Peru but restrictions apply to the availability of these data, which were used under license for the current study, and are not publicly available. Data are however available from the authors upon reasonable request and with permission of Kantar WorldPanel Peru.

## Abbreviations

APEIM: Peruvian Association of Market Research Firms (*Asociación Peruana de Empresas de Inteligencia de Mercados*)
ENAHO: Peruvian National Household Survey (*Encuesta Nacional de Hogares*)
LMIC: Low-and middle-income country
NFP: Nutrition Facts Panel
RTD: Ready-to-Drink
SES: Socio-economic status
SSB: Sugar-sweetened beverage
