# Supplementary material for: Sugar-sweetened beverage purchases in urban Peru before the implementation of taxation and warning label policies: a baseline study
Source: BMC Public Health. 2022 Dec 20;22:2389. doi: 10.1186/s12889-022-14762-w (PMC9764463; doi:10.1186/s12889-022-14762-w)
Supplement: Supplementary file 1 — Additional file 1: Supplemental Fig. 1. Timeline of the Implementation of Nutrition-Related Polices and Modifications. Supplemental Table 1. Beverage Types by Tax Status (under the 2018 regulation). Supplemental Table 2. Weighted Percentage of Households Purchasing Any Beverage by Tax Status in a Month. Supplemental Table 3. Unweighted Mean Monthly Purchase Volume by Beverage Tax Status (Liters per Capita per Household). Supplemental Table 4. Weighted Mean Monthly Purchase Volume by Beverage Tax Status (Liters per Capita per Household) in 2016. Supplemental Table 5. Weighted Mean Monthly Purchase Volume of the Top Three Beverage Types by Taxation Status (Liters per Capita per Household) in 2016. Supplemental Table 6. The Three Highest Volume Beverage Types by Region, SES, and Education in 2016. Supplemental Table 7. Weighted Mean Monthly Purchase Volume by Beverage Tax Status in Liters per Capitaa per Household (Excluding Children Aged < 2 Years). Supplemental Table 8. Weighted Mean Monthly Purchase Volume by Beverage Tax Status in Liters per Capitaa per Household (Excluding Children Aged < 5 Years). [file 12889_2022_14762_MOESM1_ESM.docx]

Supplemental Figure 1.

*
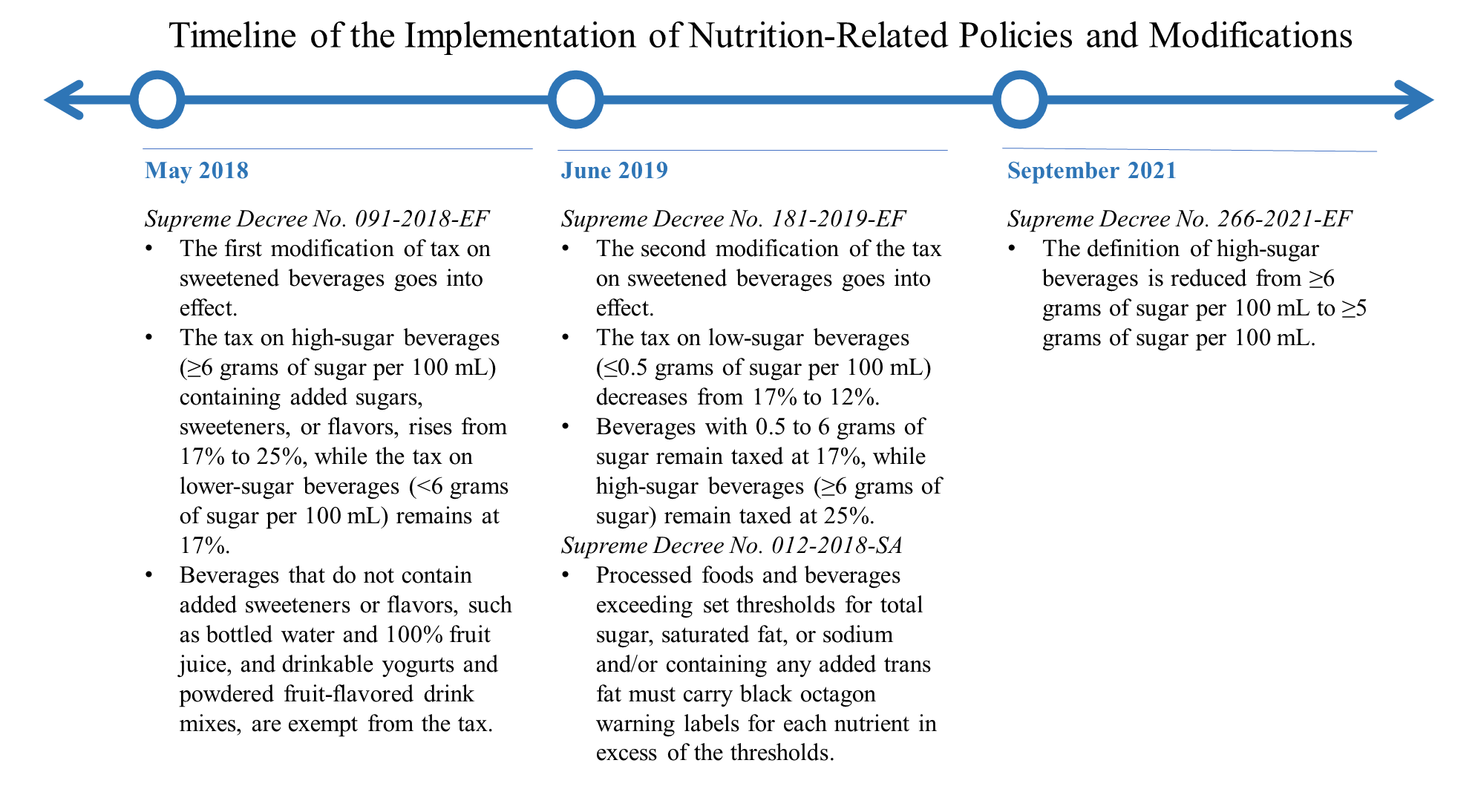
*

Supplemental Table 1. Beverage Types by Tax Status (under the 2018 regulation)

| **Untaxed Beverages**  **(No added sugar/sweetener/ flavor or exempt)** | **Lower-Sugar Taxed Beverages (<6 g/100 mL)*** | **High-Sugar Taxed Beverages (≥6 g/100 mL)*** |
| --- | --- | --- |
| Dairy drinks^a^ (powdered/ evaporated)  Fruit juice, 100%  Infant formula  Milk, plain  Milk (powdered, including flavored powdered milks)  *Refrescos*^b^ (powdered)  Water, no added flavor or sweeteners  Yogurt, drinkable | Fruit juice drinks^c^ (<100% fruit juice)  Milk, flavored or sweetened (RTD)  Soda, diet  Soda, regular  Sports drinks  Teas/coffees (RTD)  Water, flavored or sweetened | Dairy drinks^a^ (RTD)  Energy drinks  Fruit juice drinks^c^ (<100% fruit juice)  Milk, flavored or sweetened (RTD)  Milk substitutes  *Refrescos*^b^ (RTD)  Soda, regular  Sports drinks  Teas/coffees (RTD)  Water, flavored or sweetened |

Abbreviations: RTD: Ready to Drink

*Drinks in these categories are taxed at the lower-sugar rate if they contain <6 grams of sugar per 100 mL. If they contain ≥6 grams of sugar per 100 mL, they are taxed at the high-sugar rate.

^a^Dairy drinks are milk-based drinks that contain other ingredients such as oil (vegetable, palm), sugar, honey, cereal, or flour.

^b^*Refrescos* are fruit-flavored drinks containing water, sugar, and flavoring.

^c^Fruit juice drinks are comprised of nectars and juices containing <100% fruit juice.

Supplemental Table 2. Weighted Percentage of Households Purchasing Any Beverage by Tax Status in a Month

|  | **Untaxed** | | **Tax Tier 1 (Lower-sugar)** | | **Tax Tier 2 (High-Sugar)** | |
| --- | --- | --- | --- | --- | --- | --- |
| **Year** | **2016** | **2017** | **2016** | **2017** | **2016** | **2017** |
| **Overall** | 99.8 | 99.7 | 48.1 | 44.4 | 94.3 | 92.1 |
| **Region** |  |  |  |  |  |  |
| Lima | 99.9 | 99.8 | 48.4 | 45.2 | 95.3 | 92.9 |
| Central Coast | 99.9 | 99.5 | 50.3 | 47.7 | 94.3 | 92.3 |
| Northern Coast | 99.7 | 99.6 | 49.1 | 44.5 | 88.5 | 86.4 |
| Southern Coast | 99.7 | 99.5 | 31.6 | 26.8 | 96.4 | 94.4 |
| Amazon | 99.8 | 99.9 | 59.9 | 58.8 | 97.0 | 96.1 |
| Highlands | 99.9 | 99.8 | 54.2 | 47.5 | 96.8 | 95.2 |
| **Education^a,b^** |  |  |  |  |  |  |
| Less than HS | 99.9 | 99.7 | 45.7 | 43.3 | 93.4 | 92.4 |
| Graduated HS | 99.9 | 99.7 | 48.1 | 43.6 | 94.4 | 92.3 |
| More than HS | 99.8 | 99.7 | 49.4 | 45.8 | 94.7 | 91.9 |
| **SES^c^** |  |  |  |  |  |  |
| A/B (High) | 99.8 | 99.7 | 52.4 | 48.0 | 95.2 | 91.6 |
| C | 99.8 | 99.7 | 46.8 | 43.2 | 94.8 | 92.8 |
| D | 99.9 | 99.8 | 45.6 | 42.4 | 93.0 | 91.3 |
| E (Low) | 99.8 | 99.7 | 51.1 | 46.5 | 94.9 | 93.1 |

Notes: Percentages of households purchasing any beverages (e.g., >0 mL) by tax status were calculated using sample weights and standard errors clustered at the household-level.

^a^ Education was grouped into three categories: did not complete high school (secondary school), graduated high school, and completed technical school, university or graduate school.

^b^ Three households were missing education in 2017.

^c^ SES was determined based on an assets index developed by the Peruvian Association of Market Research Firms (APEIM). Categories A and B were combined because few households were in the highest SES (A) category (2016: 4.5%; 2017: 4.4%).

Supplemental Table 3. Unweighted Mean Monthly Purchase Volume by Beverage Tax Status (Liters per Capita per Household)

| **Beverage Type** | **Untaxed**  Mean (95% CI) | | **Tax Tier 1 (Lower-Sugar)**  Mean (95% CI) | | **Tax Tier 2 (High-Sugar)**  Mean (95% CI) | |
| --- | --- | --- | --- | --- | --- | --- |
|  | 2016 | 2017 | 2016 | 2017 | 2016 | 2017 |
| **Overall** | 9.9 (9.7, 10.2) | 9.5 (9.3, 9.8) | 0.3 (0.3, 0.3) | 0.3 (0.3, 0.3) | 3.0 (2.9, 3.1) | 2.8 (2.7, 2.8) |
| **Region** |  |  |  |  |  |  |
| Lima | 9.4 (9.0, 9.7) | 9.0 (8.6, 9.3) | 0.3 (0.3, 0.3) | 0.3 (0.3, 0.3) | 3.0 (2.9, 3.1) | 2.7 (2.6, 2.9) |
| Central Coast | 9.0 (8.4, 9.5) | 8.4 (7.9, 8.9) | 0.4 (0.3, 0.4) | 0.4 (0.3, 0.4) | 3.1 (2.9, 3.4) | 3.0 (2.7, 3.3) |
| Northern Coast | 8.2 (7.9, 8.6) | 8.0 (7.6, 8.4) | 0.4 (0.3, 0.4) | 0.3 (0.3, 0.4) | 1.8 (1.7, 1.9) | 1.7 (1.6, 1.8) |
| Southern Coast | 9.0 (8.5, 9.5) | 8.4 (7.9, 8.9) | 0.2 (0.2, 0.2) | 0.2 (0.1, 0.2) | 4.2 (3.9, 4.4) | 3.5 (3.3, 3.8) |
| Amazon | 19.3 (17.4, 21.2) | 20.5 (18.5, 22.5) | 0.3 (0.3, 0.4) | 0.3 (0.3, 0.4) | 3.0 (2.8, 3.2) | 2.8 (2.6, 3.0) |
| Highlands | 10.4 (9.8, 11.1) | 9.3 (8.7, 9.9) | 0.4 (0.3, 0.4) | 0.3 (0.3, 0.4) | 4.5 (4.1, 4.8) | 3.9 (3.6, 4.2) |
| **Education^a,b^** |  |  |  |  |  |  |
| Less than HS | 8.6 (8.1, 9.2) | 8.0 (7.5, 8.5) | 0.3 (0.3, 0.3) | 0.3 (0.3, 0.3) | 2.8 (2.6, 2.9) | 2.6 (2.4, 2.8) |
| HS Graduate | 9.0 (8.6, 9.3) | 8.6 (8.3, 9.0) | 0.3 (0.3, 0.3) | 0.3 (0.3, 0.3) | 2.8 (2.7, 2.9) | 2.6 (2.5, 2.7) |
| More than HS | 11.6 (11.2, 12.1) | 11.4 (10.9, 11.9) | 0.4 (0.3, 0.4) | 0.3 (0.3, 0.4) | 3.4 (3.2, 3.5) | 3.0 (2.8, 3.1) |
| **SES^c^** |  |  |  |  |  |  |
| A/B (High) | 13.3 (12.5, 14.0) | 13.0 (12.2, 13.7) | 0.4 (0.4, 0.4) | 0.4 (0.3, 0.4) | 3.7 (3.5, 4.0) | 3.3 (3.1, 3.5) |
| C | 10.6 (10.1, 11.0) | 10.4 (9.9, 10.8) | 0.3 (0.3, 0.4) | 0.3 (0.3, 0.3) | 3.1 (2.9, 3.2) | 2.8 (2.7, 3.0) |
| D | 8.6 (8.2, 9.0) | 8.0 (7.6, 8.4) | 0.3 (0.3, 0.3) | 0.3 (0.3, 0.3) | 2.7 (2.6, 2.8) | 2.5 (2.4, 2.6) |
| E (Low) | 7.2 (6.6, 7.8) | 6.9 (6.3, 7.4) | 0.3 (0.3, 0.3) | 0.3 (0.3, 0.3) | 2.7 (2.5, 2.9) | 2.5 (2.3, 2.7) |

Notes: Mean volume by tax status, overall and by key demographic characteristics, was calculated using standard errors clustered at the household-level.

^a^ Education was grouped into three categories: did not complete high school (secondary school), graduated high school, and completed technical school, university or graduate school.

^b^ Three households were missing education in 2017.

^c^ SES was determined based on an assets index developed by the Peruvian Association of Market Research Firms (APEIM). Categories A and B were combined because few households were in the highest SES (A) category (2016: 4.5%; 2017: 4.4%).

Supplemental Table 4. Weighted Mean Monthly Purchase Volume by Beverage Tax Status (Liters per Capita per Household) in 2016

| **Beverage Type** | **Untaxed** | **Tax Tier 1 (Lower-Sugar)** | **Tax Tier 2 (High** |
| --- | --- | --- | --- |
|  | Mean (95% CI) | Mean (95% CI) | Mean (95% CI) |
| **Overall** | 9.8 (9.5, 10.0) | 0.3 (0.3, 0.4) | 3.1 (3.0, 3.2) |
| **Region** |  |  |  |
| Lima | 10.1 (9.7, 10.5) | 0.3 (0.3, 0.4) | 3.2 (3.0, 3.4) |
| Central Coast | 8.9 (8.3, 9.4) | 0.4 (0.3, 0.4) | 3.1 (2.9, 3.4) |
| Northern Coast | 8.0 (7.6, 8.4) | 0.4 (0.3, 0.4) | 1.7 (1.6, 1.8) |
| Southern Coast | 8.7 (8.2, 9.2) | 0.2 (0.1, 0.2) | 4.0 (3.7, 4.2) |
| Amazon | 18.2 (15.6, 20.7) | 0.4 (0.3, 0.4) | 3.1 (2.9, 3.4) |
| Highlands | 9.5 (8.7, 10.4) | 0.4 (0.3, 0.5) | 4.3 (3.8, 4.8) |
| **Education^a^** |  |  |  |
| Less than HS | 8.8 (8.1, 9.4) | 0.3 (0.3, 0.3) | 2.8 (2.6, 3.0) |
| HS Graduate | 8.9 (8.5, 9.3) | 0.3 (0.3, 0.3) | 2.9 (2.7, 3.0) |
| More than HS | 11.2 (10.8, 11.7) | 0.4 (0.3, 0.4) | 3.5 (3.3, 3.7) |
| **SES^b^** |  |  |  |
| A/B (High) | 13.2 (12.4, 14.0) | 0.4 (0.4, 0.5) | 4.1 (3.7, 4.5) |
| C | 9.9 (9.5, 10.3) | 0.3 (0.3, 0.3) | 3.1 (2.9, 3.2) |
| D | 8.6 (8.1, 9.0) | 0.3 (0.3, 0.3) | 2.6 (2.5, 2.7) |
| E (Low) | 7.3 (6.6, 8.1) | 0.3 (0.3, 0.4) | 2.8 (2.5, 3.1) |

Notes: Mean volume by tax status, overall and by key demographic characteristics, was calculated using sample weights and standard errors clustered at the household-level.

^a^ Education was grouped into three categories: did not complete high school (secondary school), graduated high school, and completed technical school, university or graduate school.

^b^ SES was determined based on an assets index developed by the Peruvian Association of Market Research Firms (APEIM). Categories A and B were combined because few households were in the highest SES (A) category (2016: 4.5%).

Supplemental Table 5. Weighted Mean Monthly Purchase Volume of the Top Three Beverage Types by Taxation Status (Liters per Capita per Household) in 2016

|  | Mean (95% CI)  L/capita/  household | Percent Purchasers (%) (95% CI) | Mean (95% CI)  L/capita/  household  (Among Purchasers) |
| --- | --- | --- | --- |
| Untaxed |  |  |  |
| *Water, plain* | 1.7 (1.5, 1.8) | 62.7 (61.5, 63.9) | 2.7 (2.5, 2.9) |
| *Milk, plain* | 1.9 (1.9, 2.0) | 85.4 (84.6, 86.2) | 2.3 (2.2, 2.3) |
| *Dairy drinks^a^* | 1.3 (1.2, 1.3) | 66.1 (64.9, 67.3) | 1.9 (1.8, 2.0) |
| Lower-sugar Taxed |  |  |  |
| *Regular soda* | 0.2 (0.1, 0.2) | 23.7 (22.8, 24.7) | 0.6 (0.6, 0.7) |
| *Diet soda* | 0.1 (0.1, 0.1) | 11.7 (11.0, 12.4) | 0.5 (0.5, 0.6) |
| *Sports drinks* | 0.1 (0.1, 0.1) | 26.2 (25.1, 27.2) | 0.4 (0.4, 0.4) |
| High-sugar Taxed | \|  \| \| --- \| |  |  |
| *Regular soda* | 2.3 (2.2, 2.4) | 88.3 (87.6, 89.0) | 2.6 (2.5, 2.7) |
| *Fruit juice drinks^b^* | 0.3 (0.3, 0.3) | 48.1 (46.9, 49.3) | 0.6 (0.5, 0.6) |
| *Refrescos^c^* | 0.2 (0.2, 0.3) | 33.1 (32.0, 34.2) | 0.7 (0.7, 0.8) |

Notes: Mean volume for the three highest volume beverage types in each tax status category was calculated using sample weights and standard errors clustered at the household-level.

^a^ Dairy drinks are milk-based drinks that contain other ingredients such as oil (vegetable, palm), sugar, honey, cereal, or flour.

^b^ Fruit juice drinks include nectars and juices containing <100% fruit juice.

^c^ *Refrescos* are fruit-flavored drinks, containing water, sugar, and flavoring.

Supplemental Table 6. The Three Highest Volume Beverage Types by Region, SES, and Education in 2016

|  | **Beverage #1** | | **Beverage #2** | | **Beverage #3** | |
| --- | --- | --- | --- | --- | --- | --- |
|  | **Bev type** | **Per capita mean (95% CI)** | **Bev type** | **Per capita mean (95% CI)** | **Bev type** | **Per capita mean (95% CI)** |
| **Total** | Soda (reg) | 2.4 (2.3, 2.5) | Milk (plain) | 1.9 (1.9, 2.0) | Water | 1.7 (1.5, 1.8) |
| **Region** |  |  |  |  |  |  |
| Lima | Soda (reg) | 2.5 (2.4, 2.7) | Milk (plain) | 1.9 (1.8, 2.0) | Dairy drinks^c^ | 1.5 (1.4, 1.6) |
| Central Coast | Soda (reg) | 2.5 (2.3, 2.7) | Milk (plain) | 2.0 (1.8, 2.2) | Water | 1.5 (1.3, 1.7) |
| Northern Coast | Milk (plain) | 1.9 (1.8, 2.0) | Soda (reg) | 1.5 (1.4, 1.6) | Water | 1.4 (1.2, 1.6) |
| Southern Coast | Soda (reg) | 3.2 (3.0, 3.4) | Milk (plain) | 1.9 (1.8, 2.1) | Dairy drinks^c^ | 1.2 (1.1, 1.3) |
| Amazon | Water | 11.7 (9.6, 13.8) | Soda (reg) | 2.7 (2.4, 2.9) | Milk (plain) | 1.2 (1.0, 1.3) |
| Highlands | Soda (reg) | 3.0 (2.7, 3.3) | Milk (plain) | 2.3 (2.1, 2.5) | Dairy drinks^c^ | 1.4 (1.2, 1.6) |
| **Education^a^** |  |  |  |  |  |  |
| Less than HS | Soda (reg) | 2.3 (2.1, 2.4) | Milk (plain) | 1.8 (1.7, 2.0) | Water | 1.5 (1.1, 1.8) |
| HS Graduate | Soda (reg) | 2.3 (2.2, 2.4) | Milk (plain) | 1.8 (1.7, 1.9) | Water | 1.5 (1.3, 1.7) |
| More than HS | Soda (reg) | 2.7 (2.5, 2.9) | Milk (plain) | 2.1 (2.0, 2.2) | Water | 2.0 (1.8, 2.2) |
| **SES^b^** |  |  |  |  |  |  |
| AB | Soda (reg) | 3.1 (2.8, 3.4) | Water | 2.6 (2.2, 3.0) | Milk (plain) | 2.3 (2.1, 2.5) |
| C | Soda (reg) | 2.5 (2.4, 2.7) | Milk (plain) | 2.0 (1.9, 2.1) | Water | 1.5 (1.3, 1.6) |
| D | Soda (reg) | 2.1 (2.0, 2.2) | Milk (plain) | 1.8 (1.7, 1.9) | Water | 1.4 (1.2, 1.6) |
| E | Soda (reg) | 2.1 (1.9, 2.3) | Water | 1.5 (1.1, 2.0) | Milk (plain) | 1.5 (1.4, 1.7) |

Notes: Mean volume for the highest volume beverage types (independent of tax status), overall and by key demographic characteristics, for 2016 was calculated using sample weights and standard errors clustered at the household-level.

^a^ Education was grouped into three categories: did not complete high school (secondary school), graduated high school, and completed technical school, university or graduate school.

^b^ SES was determined based on an assets index developed by the Peruvian Association of Market Research Firms (APEIM). Categories A and B were combined because few households were in the highest SES (A) category (4.5%).

^c^ Dairy drinks are milk-based drinks that contain other ingredients such as oil (vegetable, palm), sugar, honey, cereal, or flour.

Supplemental Table 7. Weighted Mean Monthly Purchase Volume by Beverage Tax Status in Liters per Capita^a^ per Household (Excluding Children Aged <2 Years)

| **Beverage Type** | **Untaxed**  Mean (95% CI) | | **Tax Tier 1 (Lower-Sugar)**  Mean (95% CI) | | **Tax Tier 2 (High-Sugar)**  Mean (95% CI) | |
| --- | --- | --- | --- | --- | --- | --- |
|  | 2016 | 2017 | 2016 | 2017 | 2016 | 2017 |
| **Overall** | 10.4 (10.1, 10.6) | 9.7 (9.4, 10.0) | 0.4 (0.3, 0.4) | 0.3 (0.3, 0.4) | 3.3 (3.2, 3.4) | 2.9 (2.8, 3.0) |
| **Region** |  |  |  |  |  |  |
| Lima | 10.8 (10.4, 11.2) | 10.1 (9.7, 10.6) | 0.4 (0.3, 0.4) | 0.4 (0.3, 0.4) | 3.4 (3.2, 3.6) | 3.1 (2.9, 3.2) |
| Central Coast | 9.3 (8.8, 9.9) | 8.5 (8.0, 9.0) | 0.4 (0.3, 0.4) | 0.4 (0.3, 0.4) | 3.3 (3.0, 3.5) | 3.1 (2.8, 3.3) |
| Northern Coast | 8.4 (8.0, 8.8) | 8.1 (7.6, 8.5) | 0.4 (0.4, 0.4) | 0.3 (0.3, 0.4) | 1.7 (1.6, 1.8) | 1.7 (1.6, 1.8) |
| Southern Coast | 9.1 (8.6, 9.6) | 8.3 (7.8, 8.8) | 0.2 (0.2, 0.2) | 0.2 (0.1, 0.2) | 4.2 (3.9, 4.4) | 3.5 (3.2, 3.7) |
| Amazon | 18.8 (16.3, 21.4) | 18.9 (16.5, 21.3) | 0.4 (0.3, 0.4) | 0.3 (0.3, 0.4) | 3.3 (3.0, 3.6) | 2.9 (2.7, 3.2) |
| Highlands | 10.1 (9.2, 11.0) | 8.6 (7.9, 9.3) | 0.4 (0.3, 0.5) | 0.3 (0.3, 0.4) | 4.6 (4.1, 5.1) | 3.9 (3.5, 4.3) |
| **Education^b,c^** |  |  |  |  |  |  |
| Less than HS | 9.3 (8.6, 9.9) | 8.4 (7.8, 9.0) | 0.3 (0.3, 0.4) | 0.3 (0.3, 0.4) | 2.9 (2.7, 3.1) | 2.8 (2.5, 3.0) |
| HS Graduate | 9.6 (9.2, 10.0) | 8.8 (8.4, 9.2) | 0.3 (0.3, 0.4) | 0.3 (0.3, 0.3) | 3.1 (2.9, 3.3) | 2.7 (2.6, 2.9) |
| More than HS | 11.8 (11.3, 12.2) | 11.5 (10.9, 12.0) | 0.4 (0.4, 0.4) | 0.4 (0.3, 0.4) | 3.7 (3.4, 3.9) | 3.2 (3.0, 3.5) |
| **SES^d^** |  |  |  |  |  |  |
| A/B (High) | 13.8 (13.0, 14.6) | 13.5 (12.6, 14.3) | 0.5 (0.4, 0.5) | 0.5 (0.4, 0.5) | 4.3 (3.9, 4.6) | 3.7 (3.3, 4.0) |
| C | 10.6 (10.2, 11.0) | 9.8 (9.4, 10.3) | 0.3 (0.3, 0.4) | 0.3 (0.3, 0.4) | 3.3 (3.1, 3.5) | 2.9 (2.8, 3.1) |
| D | 9.1 (8.7, 9.6) | 8.4 (8.0, 8.8) | 0.3 (0.3, 0.3) | 0.3 (0.3, 0.3) | 2.8 (2.6, 2.9) | 2.6 (2.4, 2.7) |
| E (Low) | 7.8 (7.1, 8.6) | 7.2 (6.6, 7.8) | 0.3 (0.3, 0.4) | 0.3 (0.3, 0.4) | 3.0 (2.6, 3.3) | 2.6 (2.4, 2.9) |

Notes: Mean volume by tax status, overall and by key demographic characteristics, was calculated using sample weights and standard errors clustered at the household-level.

^a^ Per-capita volume was calculated by dividing household purchase volume by the number of household members, excluding children under the age of 2.

^b^ Education was grouped into three categories: did not complete high school (secondary school), graduated high school, and completed technical school, university or graduate school.

^c^ Three households were missing education in 2017.

^d^ SES was determined based on an assets index developed by the Peruvian Association of Market Research Firms (APEIM). Categories A and B were combined because few households were in the highest SES (A) category (2016: 4.5%; 2017: 4.4%).

Supplemental Table 8. Weighted Mean Monthly Purchase Volume by Beverage Tax Status in Liters per Capita^a^ per Household (Excluding Children Aged <5 Years)

| **Beverage Type** | **Untaxed**  Mean (95% CI) | | **Tax Tier 1 (Lower-Sugar)**  Mean (95% CI) | | **Tax Tier 2 (High-Sugar)**  Mean (95% CI) | |
| --- | --- | --- | --- | --- | --- | --- |
|  | 2016 | 2017 | 2016 | 2017 | 2016 | 2017 |
| **Overall** | 11.3 (11.0, 11.6) | 10.5 (10.3, 10.8) | 0.4 (0.4, 0.4) | 0.4 (0.3, 0.4) | 3.6 (3.4, 3.7) | 3.2 (3.1, 3.3) |
| **Region** |  |  |  |  |  |  |
| Lima | 11.8 (11.3, 12.2) | 11.1 (10.7, 11.6) | 0.4 (0.4, 0.4) | 0.4 (0.4, 0.4) | 3.7 (3.5, 3.9) | 3.4 (3.2, 3.6) |
| Central Coast | 10.2 (9.6, 10.9) | 9.2 (8.7, 9.8) | 0.4 (0.4, 0.5) | 0.4 (0.3, 0.5) | 3.6 (3.3, 3.9) | 3.3 (3.1, 3.6) |
| Northern Coast | 9.1 (8.6, 9.5) | 8.6 (8.2, 9.1) | 0.4 (0.4, 0.5) | 0.4 (0.3, 0.4) | 1.9 (1.8, 2.0) | 1.8 (1.7, 1.9) |
| Southern Coast | 9.9 (9.4, 10.5) | 8.9 (8.4, 9.4) | 0.2 (0.2, 0.2) | 0.2 (0.1, 0.2) | 4.5 (4.2, 4.8) | 3.7 (3.5, 4.0) |
| Amazon | 20.3 (17.3, 23.2) | 20.2 (17.6, 22.7) | 0.4 (0.3, 0.5) | 0.4 (0.3, 0.4) | 3.5 (3.2, 3.8) | 3.2 (2.9, 3.5) |
| Highlands | 11.0 (10.1, 12.0) | 9.3 (8.6, 10.0) | 0.4 (0.4, 0.5) | 0.4 (0.3, 0.4) | 5.0 (4.4, 5.5) | 4.2 (3.8, 4.6) |
| **Education^b,c^** |  |  |  |  |  |  |
| Less than HS | 9.9 (9.2, 10.5) | 8.9 (8.3, 9.5) | 0.3 (0.3, 0.4) | 0.3 (0.3, 0.4) | 3.1 (2.9, 3.3) | 3.0 (2.7, 3.2) |
| HS Graduate | 10.6 (10.2, 11.1) | 9.8 (9.4, 10.2) | 0.4 (0.3, 0.4) | 0.3 (0.3, 0.4) | 3.4 (3.2, 3.6) | 3.0 (2.9, 3.2) |
| More than HS | 12.7 (12.3, 13.2) | 12.3 (11.8, 12.9) | 0.4 (0.4, 0.5) | 0.4 (0.4, 0.5) | 4.0 (3.7, 4.2) | 3.5 (3.3, 3.7) |
| **SES^d^** |  |  |  |  |  |  |
| A/B (High) | 14.7 (13.8, 15.5) | 14.1 (13.3, 14.9) | 0.5 (0.4, 0.5) | 0.5 (0.4, 0.5) | 4.5 (4.2, 4.9) | 3.9 (3.5, 4.2) |
| C | 11.5 (11.1, 11.9) | 10.9 (10.5, 11.4) | 0.4 (0.3, 0.4) | 0.4 (0.3, 0.4) | 3.6 (3.4, 3.8) | 3.3 (3.1, 3.5) |
| D | 10.1 (9.6, 10.5) | 9.1 (8.7, 9.6) | 0.3 (0.3, 0.4) | 0.3 (0.3, 0.3) | 3.1 (2.9, 3.2) | 2.8 (2.6, 2.9) |
| E (Low) | 8.7 (7.9, 9.6) | 7.8 (7.2, 8.4) | 0.4 (0.3, 0.4) | 0.3 (0.3, 0.4) | 3.2 (2.9, 3.6) | 2.9 (2.6, 3.1) |

Notes: Mean volume by tax status, overall and by key demographic characteristics, was calculated using sample weights and standard errors clustered at the household-level.

^a^ Per-capita volume was calculated by dividing household purchase volume by the number of household members, excluding children under the age of 5.

^b^ Education was grouped into three categories: did not complete high school (secondary school), graduated high school, and completed technical school, university or graduate school.

^c^ Three households were missing education in 2017.

^d^ SES was determined based on an assets index developed by the Peruvian Association of Market Research Firms (APEIM). Categories A and B were combined because few households were in the highest SES (A) category (2016: 4.5%; 2017: 4.4%).
